# Self-Reported Prevalence of Chronic Non-Communicable Diseases in Relation to Socioeconomic and Educational Factors in Colombia: A Community-Based Study in 11 Departments

**DOI:** 10.5334/gh.792

**Published:** 2020-04-21

**Authors:** Paul A. Camacho, Diego Gomez-Arbelaez, Johanna Otero, Silvia González-Gómez, Dora I. Molina, Gregorio Sanchez, Edgar Arcos, Claudia Narvaez, Henry García, Maritza Pérez, Eric Hernandez-Triana, Myriam Duran, Carlos Cure, Aristides Sotomayor, Alvaro Rico, Fresia Cotes, Sumathy Rangarajan, Salim Yusuf, Patricio López-Jaramillo

**Affiliations:** 1 Fundación Oftalmológica de Santander—FOSCAL, Floridablanca, CO; 2 Universidad Autónoma de Bucaramanga, Bucaramanga, CO; 3 Universidad de Santander, Bucaramanga, CO; 4 Universidad de Caldas y Médicos Internistas de Caldas, Manizales, CO; 5 Universidad del Quindío, Hospital San Juan de Dios, Armenia, CO; 6 Fundación Cometa, Pasto, CO; 7 Hospital Susana López, Popayán, CO; 8 Fundación RIESCAR, El Espinal, CO; 9 Facultad de Medicina, Universidad Militar Nueva Granada, Bogotá, CO; 10 ENDOCARE, Bogotá, CO; 11 BIOMELAB Research Center, CO; 12 Centro Cardiovascular Santa Lucia, Cartagena, CO; 13 FINDEMOS, Yopal, CO; 14 Universidad de Santander, Valledupar, CO; 15 PHRI, McMaster University, Hamilton, CA; 16 Facultad de Ciencias de la Salud Eugenio Espejo, UTE, Quito, EC

**Keywords:** chronic non-communicable diseases, self-reported prevalence, Colombia

## Abstract

**Background:**

Chronic non-communicable diseases are prevalent conditions in developing countries, such as Colombia. Several socioeconomic and educational factors have been associated with these pathologies. However, there is little country-specific information regarding the self-reported prevalence of chronic diseases and their association with the aforementioned factors in Colombia.

**Objectives:**

To evaluate the current situation of chronic non-transmissible diseases in Colombia by self-report and to analyze its potential relationship with sociodemographic, economic and educational factors.

**Methods:**

This is a cross-sectional baseline sub-analysis from the prospective, standardized collaborative PURE study in Colombia. Participants were recruited between 2005 to 2009, in 11 departments of the country, and included 7,485 subjects of 35 to 70 years old. Questionnaires of self-reported chronic non-communicable diseases, and demographic, socioeconomic and educational variables were applied.

**Results:**

Hypertension was the most prevalent chronic condition reported with a prevalence of 22.2% (21.2%–23.1%, 95% CI), followed by diabetes with a prevalence of 5.7% (5.1%–6.2%, 95% CI), asthma 2.7% (2.2%–3.0%, 95% CI), coronary heart disease 2.4% (2.0%–2.7%, 95% CI), stroke and heart failure 1.5% (1.2%–1.8%, 95% CI) each, chronic obstructive pulmonary disease 1.2% (0.6%–1.5%, 95% CI), and cancer 1.2% (1.0%–1.5%, 95% CI). Among the study sample, 23.3% (22.4%–24.3%, 95% CI) reported having one chronic NCDs, and 6.4% (5.9%–7.0%, 95% CI) reported having multiple chronic NCDs. The prevalence of multiple NCDs increased significantly with age, was more common in those from households with higher income, whereas it was significantly lower in persons with high education.

The central and central-east regions of the country are those with the higher prevalence of self-reported NCDs.

**Conclusion:**

The results of the current study indicate the presence of socioeconomic and educational inequalities in the distribution of chronic NCDs in the Colombian population.

## Introduction

The burden of chronic non-communicable diseases (NCDs) is growing worldwide, but especially in developing countries such as Colombia [1]. According to the World Health Organization (WHO) the chronic NCDs, including cardiovascular diseases, stroke, cancer, diabetes mellitus and chronic respiratory diseases, are responsible for almost 70% of deaths worldwide [2] and account for roughly 38 million deaths per year [3]. Almost 80% of all these chronic NCDs-related deaths, especially those at younger ages occur in developing countries [4]. Hence, the impact of chronic NCDs is far-reaching with potentially serious consequences not only for the health of the population but also for the economy [5,6].

In Colombia chronic NCDs are considered the main cause of morbidity and mortality. There were 178,000 deaths from NCDs between 2014 and 2017 which accounts for 73% of total deaths during this time period [7]. An increase of 22.7% in the total deaths of chronic NCDs was estimated to have occurred between 2007 and 2017 [1]. Several factors are thought to have influenced this epidemiological trend, including rapid urbanization, migration from rural to urban areas, changes in lifestyles and nutritional patterns, lack of health-related awareness, low education and low socioeconomic status [8,9]. Moreover, social inequities have been reported as an important factor for chronic NCDs in different populations [10,11]. In Colombia, we have previously reported that the rates of awareness, treatment and control of hypertension are strongly influenced by social disparities [12]. However, information about the educational and economic factors that influence NCDs in Colombia is limited.

The present paper aims to describe the association between socioeconomic and educational characteristics of the Colombian population included in the Prospective Urban Rural Epidemiology (PURE) study and the prevalence of self-reported chronic NCDs.

## Methods

### Study design and participants

The methods and population characteristics of the PURE study have been described previously [12]. In Colombia, the Ethics Committee of the Cardiovascular Foundation of Colombia approved the study. With the purpose of obtaining an adequate geographical and social representation of the country, participants were selected from urban and rural communities from 11 of the most populated departments (states) [13]. The study sample was recruited from 2005 to 2009. A multistage convenience sample survey was used. During the first and second stages, the departments and communities were selected. In the third stage, a representative sample of households was recruited, using a community-sampling framework. The primary sample unit was the community defined as a geographical area, in which a group of people with common characteristics lived. The urban area was defined as the capital city of each department together with its metropolitan area. Households were defined and selected as part of the rural sample if located at least 50 km from an urban center, had a population of 5,000 inhabitants or less, and were at least to 45 minutes of distance from a local referral hospital.

In general, households were eligible if at least one member was 35–70 years old and if the members intended to continue living at their current address for the next four years. All eligible individuals who provided written informed consent were enrolled.

### Assessment of chronic non-communicable diseases

The outcome variable was the self-reported diagnosis of chronic NCDs. Participants were asked whether they had ever been told by a physician that they had any of the following health conditions: hypertension (HTN), diabetes mellitus (DM), coronary heart disease (CHD), stroke, heart failure (HF), chronic obstructive pulmonary disease (COPD), asthma and cancer.

### Socioeconomic and educational characteristics

Demographic, socioeconomic, and educational variables were included in the analyses as risk factors for self-reported chronic NCDs. Demographic factors included: sex (male or female), age, marital status, residence area (rural or urban) and department (state) of residence. Age was categorized into three age categories: 1) ≤49, 2) 50–59, and 3) ≥60. Marital status was recoded into three categories: 1) single, 2) married or free union, and 3) separated or divorced or widowed. In order to do a geographical assessment, departments (states) were recoded into four categories: 1) Atlantic region (Atlántico, Bolívar and Cesar), 2) Central region (Caldas, Quindío and Tolima), 3) Central-East region (Santander, Casanare and Cundinamarca), and 4) Pacific region (Cauca and Nariño).

Socioeconomic factors included: education level and household income. Education level was recoded into three categories: 1) no education, 2) ≤11 years of education, and 3) >11 years of education. At the time of recruitment, the Colombian minimum monthly wage (MMW) was approximately 175 USD. In this analysis, the household incomes were categorized as: 1) low-to-middle income (1 to 2 MMW, <350 USD), and 2) middle income (≥2 MMW, ≥350 USD).

As a part of the educational aspect and to evaluate the treatment awareness and compliance, a further question was asked regarding whether the participants had been taking any medications for any of the aforementioned chronic NCDs in the past month.

### Statistical Considerations

Data were entered into the iDATAfax system (Clinical Datafax Systems Inc, Hamilton, Ontario, Canada). Data analysis was performed using STATA/SE (Stata Statistics Software: Release 12 StataCorp LP, College Station, Texas, USA). A descriptive analysis estimating the measures of central tendency and dispersion was carried out. Categorical variables are presented as frequencies and percentages. For the prevalence of each chronic NCDs, the 95% confidence interval is also reported. Continuous variables are presented as means and standard deviations. Results are presented in contingency tables, and Pearson’s chi-squared tests for the prevalence and associated factors of chronic NCDs were conducted. Then, we conducted an exploratory multivariate analysis through a no conditional logistic regression to assess the demographic and social influence on the presence of chronic noncommunicable diseases. Statistical significance was set at p < 0.05 for a two-tailed test.

## Results

### Relationship between self-reported chronic NCDs and socioeconomic and educational factors

A total of 7,485 individuals aged 35 to 70 years were recruited in the PURE study in Colombia. At baseline, participants had a mean age of 50.7 ± 9.61 years, 64.1% were women and 49.5% of the population belongs to rural areas. Other baseline characteristics are presented in Table 1.

**Table 1:** Relationship between self-reported chronic non-communicable diseases and sociodemographic factors in Colombia.

	Self-reported chronic NCDs							

	HTN	DM	CHD	Stroke	HF	COPD	Asthma	Cancer

Total	1661 (22.2)	422 (5.7)	178 (2.4)	109 (1.5)	113 (1.5)	90 (1.2)	198 (2.7)	93 (1.2)
95% CI	[21.2–23.1]	[5.1–6.2]	[2.0–2.7]	[1.2–1.8]	[1.2–1.8]	[0.6–1.5]	[2.2–3.0]	[1.0–1.5]
Sex								
Female	1200 (25.2)	285 (6.0)	113 (2.4)	79 (1.7)	77 (1.6)	61 (1.3)	149 (3.1)	79 (1.7)
Male	454 (17.0)	137 (5.1)	65 (2.4)	30 (1.1)	36 (1.3)	29 (1.1)	49 (1.8)	14 (0.5)
Age (years)								
≤49	369 (11.2)	106 (3.2)	44 (1.3)	32 (1.0)	28 (0.9)	24 (0.7)	81 (2.5)	30 (0.9)
50–59	527 (26.5)	149 (7.5)	56 (2.8)	31 (1.6)	25 (1.3)	24 (1.2)	50 (2.5)	18 (0.9)
≥60	572 (39.0)	119 (8.1)	59 (4.0)	37 (2.5)	51 (3.5)	32 (2.2)	44 (3.0)	32 (2.2)
Education (years)								
None	422 (25.5)	119 (7.2)	35 (2.1)	22 (1.3)	21 (1.3)	35 (2.1)	41 (2.5)	13 (0.8)
≤11	1034 (21.8)	254 (5.3)	112 (2.4)	66 (1.4)	70 (1.5)	49 (1.0)	130 (2.7)	56 (1.2)
>11	203 (19.1)	48 (4.5)	31 (2.9)	21 (2.0)	21 (2.0)	6 (0.6)	27 (2.5)	24 (2.3)
Marital status								
Single	183 (19.0)	40 (4.2)	22 (2.3)	18 (1.9)	11 (1.1)	7 (0.7)	28 (2.9)	17 (1.8)
Married/Free union	1130 (21.2)	299 (5.6)	123 (2.3)	66 (1.2)	75 (1.4)	69 (1.3)	136 (2.6)	56 (1.1)
Separated/Divorced/Widowed	340 (29.1)	82 (7.0)	32 (2.7)	25 (2.1)	26 (2.2)	14 (1.2)	34 (2.9)	20 (1.7)
Household income								
<350 USD	1049 (21.2)	279 (5.6)	106 (2.1)	69 (1.4)	64 (1.3)	67 (1.4)	123 (2.5)	49 (1.0)
≥350 USD	612 (24.1)	143 (5.6)	72 (2.8)	40 (1.6)	49 (1.9)	23 (0.9)	75 (3.0)	44 (1.7)
Residence area								
Rural	733 (19.7)	190 (5.1)	62 (1.7)	36 (1.0)	31 (0.8)	51 (1.4)	85 (2.3)	29 (0.8)
Urban	928 (24.6)	232 (6.1)	116 (3.1)	75 (1.9)	82 (2.2)	39 (1.0)	113 (3.0)	64 (1.7)

Data are presented as count (percentage). NCDs: non-communicable chronic diseases; HTN: hypertension; DM: mellitus diabetes; CHD: coronary heart disease; HF: heart failure; COPD: chronic obstructive pulmonary disease.

HTN was the most prevalent chronic NCDs reported across the sample with a prevalence of 22.2% (21.2%–23.1%, 95% CI), followed by DM with 5.7% (5.1%–6.2%, 95% CI), and asthma with 2.7% (2.2%–3.0%, 95% CI) (Table 1). Cancer and COPD had the lowest prevalence and affected 1.2% (1.0%–1.5%, 95% CI) and 1.2% (0.6%–1.5%, 95% CI) of the study sample, respectively (Table 1). Meanwhile, a 2.4% (2.0%–2.7%, 95% CI) of the study sample reported having CHD, 1.5% (1.2%–1.8%, 95% CI) reported having had a prior stroke, and a 1.5% (1.2%–1.8%, 95% CI) reported having HF (Table 1).

The prevalence of all chronic NCDs was higher among women, except for CHD that was similar in both sexes, and in the older participants (≥60 years old). Low education was associated with a higher HTN, DM and COPD, whereas CHD, stroke, HF and cancer were common in those with >11 years of education. All chronic NCDs, except DM and COPD, had a higher prevalence in the highest income group. Those participants that were separated, divorced or widowed, and residents of urban areas also had the higher prevalence of most of the chronic NCDs (Table 1).

### Relationship between self-reported chronic NCDs multimorbidity and socioeconomic and educational factors

Among the study sample, 23.3% (22.4%–24.3%, 95% CI) reported having one chronic NCDs, and 6.4% (5.9%–7.0%, 95% CI) reported two or more chronic NCDs. The prevalence of NCDs multimorbidity (≥2 NCDs) was higher among women than in men (7.3% vs. 5.0%, respectively), and in participants from urban than rural areas (7.7% vs. 5.2%, respectively). The prevalence of chronic NCDs multimorbidity increased significantly with age, from 2.8% in <50 years old to 12.8% in ≥60 years old, but decreased significantly according to education level, from 7.2% among those participants without study to 6.1% in participants with >11 years of education. Single participants had a prevalence of 6.1%, married participants had a prevalence of 5.9%, whereas those participants that were separated or divorced or widowed had a prevalence of 8.9% (Table 2).

**Table 2:** Relationship between the number of self-reported chronic non-communicable diseases and sociodemographic factors in Colombia.

	Number of self-reported chronic NCDs			

	0	1	≥2	P value*

Total	5228 (69.9)	1746 (23.3)	482 (6.4)	
95% CI	[68.8–70.9]	[22.4–24.3]	[5.9–7.0]	
Sex				<0.001
Female	3197 (66.6)	1239 (25.8)	349 (7.3)	
Male	2031 (75.7)	507 (18.9)	133 (5.0)	
Age (years)				<0.001
≤49	2693 (81.5)	504 (15.3)	93 (2.8)	
50–59	1290 (64.9)	545 (27.4)	145 (7.3)	
≥60	772 (52.6)	501 (34.2)	187 (12.8)	
Education (years)				0.041
None	1094 (66.2)	437 (26.4)	118 (7.2)	
≤11	3357 (70.6)	1077 (22.7)	298 (6.3)	
>11	767 (72.0)	230 (21.6)	65 (6.1)	
Marital status				<0.001
Single	708 (73.7)	190 (19.8)	59 (6.1)	
Married/Free union	3765 (70.7)	1221 (22.9)	317 (5.9)	
Separated/Divorced/Widowed	734 (62.9)	328 (28.1)	104 (8.9)	
Household income				<0.001
<350 USD	3533 (71.5)	1074 (21.7)	314 (6.4)	
≥350 USD	1695 (66.7)	672 (26.4)	168 (6.6)	
Residence area				<0.001
Rural	2713 (73.2)	789 (21.3)	191 (5.2)	
Urban	2515 (66.6)	957 (25.3)	291 (7.7)	

Data are presented as count (percentage). NCDs: non-communicable chronic diseases. * Pearson’s chi-squared test, p < 0.05.

### Prevalence of self-reported chronic non-communicable diseases by regions

The region with the highest prevalence for all chronic NCDs, except HTN, was the Central-East region, whereas in the Pacific region there were the lowest prevalence of HTN, DM, HF, COPD and asthma (Table 3).

**Table 3:** Sociodemographic characteristics of the population and prevalence of self-reported chronic non-communicable diseases by regions in Colombia.

Region	N	Rural	Female^¥^	Age*	HTN^¥^	DM	CHD^¥^	Stroke^¥^	HF^¥^	COPD^¥^	Asthma	Cancer^¥^

Atlantic	1,794	897 (50.0)	1,192 (66.5)	50.5 ± 9.52	370 (20.7)	89 (5.0)	24 (1.3)	18 (1.0)	10 (1.6)	8 (0.5)	49 (2.7)	11 (0.6)
Central	1,819	868 (47.7)	1,204 (66.2)	51.0 ± 9.30	448 (24.6)	109 (6.0)	53 (2.9)	23 (1.3)	31 (1.7)	13 (0.7)	49 (2.7)	27 (1.5)
Central-East	2,693	1,353 (50.2)	1,656 (61.5)	50.6 ± 9.87	637 (23.7)	166 (6.2)	83 (3.1)	54 (2.0)	55 (2.0)	66 (2.5)	72 (2.7)	42 (1.6)
Pacific	1,179	591 (50.1)	747 (63.4)	50.9 ± 9.52	207 (17.5)	58 (4.9)	18 (1.5)	14 (1.2)	17 (1.5)	3 (0.3)	27 (2.3)	13 (1.1)
Total	7,485	3,709 (49.6)	4,799 (64.1)	50.7 ± 9.61	1,662 (22.2)	422 (5.7)	178 (2.4)	109 (1.5)	113 (1.5)	90 (1.2)	198 (2.7)	94 (1.2)

¥ Pearson Chi squared Test, p < 0.05. Data are presented as count (percentage) or mean ± standard deviation (*). N: number; HTN: hypertension; DM: diabetes mellitus; CHD: coronary heart disease; HF: heart failure; COPD: chronic obstructive pulmonary disease.

### Relationship between chronic NCDs and use of medications

Table 4 show rates of drug use for each chronic NCDs by regions, and the rates of disease-specific drugs used according to each self-reported chronic NCDs. The overall rate of self-reported antihypertensive drugs use was 17.0%, while the overall prevalence of self-reported HTN was 22.2%; thus, the use of self-reported antihypertensive medications in those with HTN was 73.8%. A similar pattern of disease-specific drug use was observed in self-reported diabetic patients. The lowest rate of self-reported drugs consumption was observed among the patients with self-reported CHD, for instance only 18.5% self-reported cholesterol-lowering drugs use. In the analysis by regions, the central-east region presented the lowest rate of disease-specific drugs use in patients with self-reported HTN and DM, whereas the pacific region had the lowest rates of self-reported medication use in asthmatic and CHD patients (Table 4).

**Table 4:** Relationship between self-reported use of drugs and self-reported chronic non-communicable diseases by regions in Colombia.

N (%)	Total	Atlantic	Central	Central-East	Pacific	P value

**Hypertension**						
**Antihypertensive drugs**	1,271/7,485 (17.0)	317/1,784 (17.7)	373/1,819 (20.5)	419/2,693 (15.6)	162/1,179 (13.7)	<0.001
**Antihypertensive drugs in hypertensive patients**	1,226/1,662 (73.8)	308/370 (83.2)	358/448 (79.9)	405/637 (63.6)	155/207 (74.9)	<0.001
**Elevated blood pressure and not-reported HTN**	1,343/5,817 (23.1)	391/1,421 (27.5)	308/1,371 (22.5)	367/2,054 (17.9)	277/971 (58.9)	<0.001
**2 Type Diabetes**						
**Diabetes drugs**	266/7,485 (3.6)	65/1,784 (3.6)	77/1,819 (4.2)	79/2,693 (2.9)	45/1,179 (3.8)	<0.001
**Diabetes drugs in diabetic patients**	260/422 (61.6)	64/89 (71.9)	75/109 (68.8)	79/166 (47.6)	42/58 (72.4)	<0.001
Elevated glucose and not-reported diabetes*	85/6,189 (1.4)	21/1,601 (1.3)	15/1,089 (1.4)	28/2,429 (1.2)	21/1,070 (2.0)	0.552
**Other NCD**						
**Asthma drugs**	56/7,485 (0.8)	14/1,784 (0.8)	14/1,819 (0.8)	25/2,693 (0.9)	3/1,179 (0.3)	0.203
**Asthma drugs in asthmatic patients**	43/119 (36.1)	12/28 (42.9)	10/36 (27.9)	18/45 (40.0)	3/10 (30.0)	0.556
**Cholesterol-lowering drugs**	291/7,485 (3.9)	45/1,784 (2.5)	108/1,819 (6.0)	106/2,693 (3.9)	32/1,179 (2.7)	<0.001
Cholesterol-lowering drugs in CHD patients**	186/1,460 (12.7)	32/347 (9.2)	64/414 (15.5)	71/513 (13.8)	19/186 (10.2)	0.041

Pearson chi squared test, p < 0.05. * 920 without data (fasting plasma glucose (FPG)). Data are presented as number of participants (percentage). N: number; HTN: hypertension. ** CHD patients: hypertension, angina/heart attack/coronary artery disease, stroke, heart failure and other heart disease. 409 without data (regular use of cholesterol lowering drug).

Moreover, among the entire study sample 1,661 (22.2%) participants reported to be hypertensive, while 1,342 (17.9%) had elevated blood pressure but did not report to have hypertension. Likewise, 85 (1.3%) participants had abnormal levels of fasting plasma glucose (i.e. ≥ 126 mg/dl) but they were not aware of their disease. In the central-east region there were the lowest levels of disease awareness, for both diabetes (1.1%) and HTN (13.6%), respectively.

Table 5 has the multivariate analysis that shows the risk factors associated with reporting and NCDs or more than 2 NCDs. Being female, being more than 60 years old, and living in an urban residence were associated with a higher risk of multi-morbidity.

**Table 5:** Multivariate analysis of the number of self-reported chronic non-communicable diseases and sociodemographic factors in Colombia.

	Number of self-reported chronic NCDs			

	Model 1		Model 2	

	1	≥2	1	≥2

**Sex**				
**Male**	Ref.	Ref.		
**Female**	1.55 [1.38–1.75]	1.67 [1.35–2.07]	1.60 [1.40–1.82]	1.75 [1.38–2.22]
**Age (years)**				
**≤49**	Ref.			
**50–59**	2.27 [1.97–2.61]	3.24 [2.48–4.24]	2.18 [1.90–2.51]	3.31 [2.52–4.36]
**≥60**	3.49 [3.01–4.05]	6.99 [5.38–9.08]	3.43 [2.94–4.00]	7.46 [5.67–9.83]
**Education (years)**				
**None**	Ref.	Ref.		
**≤11**	0.80 [0.71–0.92]	0.82 [0.66–1.03]	0.84 [0.72–0.98]	1.05 [0.80–1.38]
**>11**	0.74 [0.62–0.90]	0.79 [0.57–1.08]	0.65 [0.51–0.82]	0.92 [0.62–1.40]
**Marital status**				
**Single**	Ref.	Ref.		
**Married/Free union**	1.22 [1.03–1.45]	1.01 [0.76–1.35]	1.26 [1.05–1.53]	1.13 [0.81–1.56]
**Separated/Divorced/Widowed**	1.69 [1.38–2.09]	1.71 [1.22–2.39]	1.26 [1.01–1.59]	1.13 [0.77–1.63]
**Household income**				
**<350 USD**	Ref.	Ref.		
**≥350 USD**	1.30 [1.16–1.45]	1.11 [0.92–1.36]	1.34 [1.15–1.57]	0.87 [0.67–1.15]
**Residence area**				
**Rural**	Ref.	Ref.		
**Urban**	1.31 [1.17–1.46]	1.64 [1.36–1.99]	1.12 [0.96–1.30]	1.55 [1.21–1.99]

Model 1: Crude OR (univariate). Model 2: Adjusted OR with: sex, age, educational level, marital status, income and location.

## Discussion

To the best of our knowledge, this is the first study assessing the self-reported prevalence of chronic NCDs and underlying socioeconomic and educational risk factors in a nationally representative sample of the adult population in Colombia. Our main findings were: 1) The overall prevalence of self-reported chronic NCDs in the country was 29.7%, HTN being the most prevalent disease (22.2%), with the central-east region most affected; 2) almost one in four adults in Colombia has at least one chronic NCD, with 6.4% having two or more chronic NCDs; 3) self-reported NCD multimorbidity was more common among women, at older ages, having no or low levels of education, being separated, divorced or widowed, having higher household incomes, and/or among those from urban areas; 4) across the regions of the country, high rates of unawareness of illness (HTN and diabetes) and low self-reported rates of medications consumption were observed, suggesting inappropriate levels of disease and treatment awareness, and low compliance among the patients with self-reported chronic NCDs.

The self-reported prevalence of chronic NCDs and multimorbidity in our study seems to be lower than that previously reported in high-income countries [14]. A study conducted in Portugal found a prevalence of 43.9%, besides being more prevalent in lower educated people [10]. The higher rates of mortality might explain this finding. However, other explanations also could be considered, including methodological differences, age of the study population, and the number of chronic NCDs assessed. In the global PURE study, we reported that, although the risk-factor burden was lowest in low-income countries, the rates of major cardiovascular disease and death were substantially higher in low-income countries than in high-income countries. Recent follow-up data show that although cardiovascular diseases are the leading global cause of mortality, this only accounts for 23% of total deaths in high-income countries vs. 41% and 43% in MIC and LIC, respectively. This means an epidemiological transition in high-income countries that is opposite to what still occurs in low- and middle-income countries [15]. Hypertension is the major contributing risk factor for cardiovascular disease; this could be explained by the existence of awareness and treatment gaps leading to poor control of NCDs. The main gaps are related to socioeconomic factors, including low access and quality of healthcare; health-related educational factors might also be determinant for our lower rates of self-reported disease prevalence. Self-reported HTN prevalence in Colombia was lower than that of self-reported in developed countries and even in other developing countries, such as India (33%) or South Africa (78%) [16].

Under- or over-estimation of chronic diseases or risk factors through self-report is a concern for epidemiological studies and surveys [18]. In the present study, underestimation was evident in the case of self-reported HTN and diabetes. The prevalence of self-reported HTN was around 20% lower in comparison with the measured prevalence of HTN [17]. Worryingly, some participants reported to have HTN but did not meet clinical criteria for HTN nor were taking any antihypertensive medication, which suggests a lack of accurate knowledge of the population about their health status. Similar patterns could be occurring with the rest of chronic NCDs. Similar to other studies [8,18], increasing age, being female, being separated, divorced or widowed, having no schooling, having a higher household income, and residing in an urban area were related to the presence of chronic conditions.

Multimorbidity has been described to be influenced by socioeconomic status (SES) and could vary by sex, age, and country [10]. However, most of the studies conducted in high–income countries have found that persons with low SES are more likely to have multimorbidity when compared to their affluent counterparts [19]. In contrast, our study shows a higher prevalence of self-reported NCDs multimorbidity among participants with higher household income. This might be due to higher access to health care and screening or increased westernized lifestyle [20]. Similarly, the higher prevalence of chronic NCDs in urban areas could be related with the industrialized environment of the cities, or a greater disease awareness due to better access to healthcare services in these areas [8,21,22]. The lack of education could affect the non-adoption of healthy lifestyles and therefore lead to a higher prevalence of NCDs. Further, in groups of low educational level, the prevalence of NCDs may be underestimated, and the burden of disease could even be higher than that reported [23].

Women reported a higher prevalence of chronic NCDs multimorbidity than men. Differential reporting might account for the gender difference in health status, but there are some other possible explanations. Women could be more sensitive and likely to report health conditions than men [24]. Furthermore, it has been described that they tend to suffer from debilitating conditions but not fatal ones, and this explains the paradox of high morbidity and less mortality among them compared to the men [25]. Besides, women usually interrelate with health services more often than men do, leading to better health-related knowledge [26]. Medications have an essential role in preventing, curing, or controlling acute and chronic diseases; however, the rates of awareness and compliance of the treatment reported here are low, as previously reported by the PURE study globally [27] and in Latin America [28].

### Limitations

This study has some strengths and a few limitations. The main strength is the large and diverse sample of the population, given its community-based design. Although not a random sample, previous analyses suggested similarities between the population characteristics in PURE and national data in age, sex, urban/rural status, and educational profiles. While there are some differences, this will unlikely affect associations between exposure and disease [29,30]. However, our data is not totally representative of the Colombian population. The study has some potential limitations. These include the cross-sectional nature of our analysis but in future incident diseases will be available. We did not include younger people (<35 years old) but considering that chronic NCDs occur mainly in older adults, our findings are still of value. The self-reported prevalence of chronic conditions is subject to recall bias and the real prevalence may in fact be higher.

### Future directions

A more considerable effort should be made to apply new strategies that intend to reduce the gaps highlighted by our results. Implementation studies should be conducted to seek strategies that reduce the gaps that do not allow NCDs control. For example, as a means to overcome the socioeconomic barriers in the control of hypertension in our health system, the Heart Outcomes Prevention Evaluation 4 study (HOPE 4) evaluated the implementation of non-physician health workers using simplified algorithms to detect new cases, free medication administration, and the involvement of family members and friends to improve treatment, adherence, and medical follow-up. The success of this intervention in the reduction of the 10-year risk score of cardiovascular disease serves as an example for future programs that could be implemented with all NCDs.

## Conclusions

The results of the current study indicate the presence of socioeconomic differences in the prevalence of chronic NCDs in Colombia. This information is of value to the government in developing public health policies that seek to reduce the burden of NCDs and allows tailoring strategies according to the particular characteristics of each region or community. Moreover, the information presented allows us to better understand gaps in treatment and awareness of various conditions. These results suggest the need for better national data that can assist in improving health education and implementing appropriate strategies to decrease the burden of chronic NCDs.

## Author Information

We confirm that this work is original and has not been published elsewhere nor is it currently under consideration for publication elsewhere. All authors have approved this study.
